# *cis*-2,5-Bis(2-fluoro-5-meth­oxy­pheneth­yl)pyrrolidinium formate

**DOI:** 10.1107/S1600536811006143

**Published:** 2011-02-26

**Authors:** Purushothama Rao Ponugoti, Narsimha Reddy Penthala, Linda P. Dwoskin, Sean Parkin, Peter A. Crooks

**Affiliations:** a Department of Pharmaceutical Sciences, College of Pharmacy, University of Kentucky, Lexington, KY 40536, USA; b Department of Chemistry, University of Kentucky, Lexington, KY 40506, USA

## Abstract

In the title compound, C_22_H_28_F_2_NO_2_^+^·CHO_2_^−^, there are three independent pyrrolidinium formate salt mol­ecules. In each cation, the central pyrrolidinium ring is not planar and the 2,5-disubstituted phenyl­ethyl groups are in equatorial positions. In the crystal, the ions are linked into a pair of chains parallel to the *c* axis by N—H⋯O hydrogen bonds between the NH group of the pyrrolidinium ring and the formate O atoms.

## Related literature

For background to the use of lobelane analogues, see: Zheng *et al.* (2005 ▶). For pyrrolidine analogues of lobelane (systematic name 2-[6-(2-hydroxy-2-phenyl-ethyl)-1-methyl-2-piperidyl]-1-phenyl-ethanone), see: Vartak *et al.* (2009 ▶).
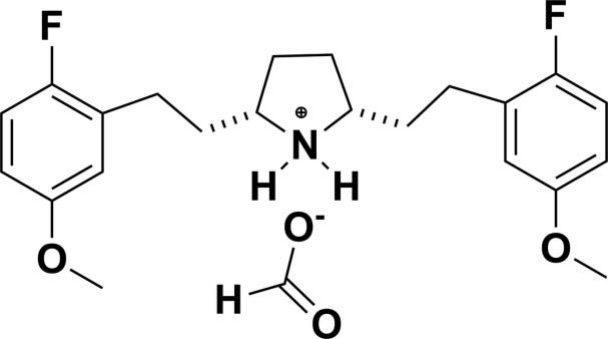


## Experimental

### 

#### Crystal data

C_22_H_28_F_2_NO_2_^+^·CHO_2_^−^
*M* *_r_* = 421.47
Orthorhombic, 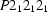

*a* = 7.8338 (1) Å
*b* = 27.8759 (3) Å
*c* = 29.3202 (3) Å
*V* = 6402.78 (13) Å^3^
*Z* = 12
Mo *K*α radiation
μ = 0.10 mm^−1^
*T* = 90 K
0.28 × 0.18 × 0.06 mm

#### Data collection

Nonius KappaCCD diffractometer
Absorption correction: multi-scan (*SCALEPACK*; Otwinowski & Minor, 1997 ▶) *T*_min_ = 0.973, *T*_max_ = 0.994
80877 measured reflections
8166 independent reflections
5100 reflections with *I* > 2σ(*I*)
*R*_int_ = 0.051

#### Refinement

*R*[*F* ^2^ > 2σ(*F* ^2^)] = 0.056
*wR*(*F* ^2^) = 0.155
*S* = 1.00
8166 reflections
817 parameters
H-atom parameters constrained
Δρ_max_ = 0.34 e Å^−3^
Δρ_min_ = −0.28 e Å^−3^

### 

Data collection: *COLLECT* (Nonius, 1998 ▶); cell refinement: *SCALEPACK* (Otwinowski & Minor, 1997 ▶); data reduction: *DENZO-SMN* (Otwinowski & Minor, 1997 ▶); program(s) used to solve structure: *SHELXS97* (Sheldrick, 2008 ▶); program(s) used to refine structure: *SHELXL97* (Sheldrick, 2008 ▶); molecular graphics: *XP* in *SHELXTL* (Sheldrick, 2008 ▶); software used to prepare material for publication: *SHELXL97* and local procedures.

## Supplementary Material

Crystal structure: contains datablocks global, I. DOI: 10.1107/S1600536811006143/hg2767sup1.cif

Structure factors: contains datablocks I. DOI: 10.1107/S1600536811006143/hg2767Isup2.hkl

Additional supplementary materials:  crystallographic information; 3D view; checkCIF report

## Figures and Tables

**Table 1:** Hydrogen-bond geometry (Å, °)

*D*—H⋯*A*	*D*—H	H⋯*A*	*D*⋯*A*	*D*—H⋯*A*
N1*A*—H1*A*1⋯O2*S*3	0.92	1.84	2.750 (4)	172
N1*A*—H1*A*2⋯O2*S*1^i^	0.92	1.83	2.737 (4)	167
N1*A*—H1*A*2⋯O1*S*1^i^	0.92	2.60	3.314 (4)	135
N1*B*—H1*B*1⋯O1*S*3	0.92	1.83	2.729 (4)	166
N1*B*—H1*B*1⋯O2*S*3	0.92	2.61	3.329 (4)	136
N1*B*—H1*B*2⋯O1*S*1	0.92	1.84	2.756 (4)	173
N1*C*—H1*C*1⋯O2*S*2^ii^	0.92	1.85	2.743 (4)	165
N1*C*—H1*C*1⋯O1*S*2^ii^	0.92	2.61	3.330 (4)	135
N1*C*—H1*C*2⋯O1*S*2	0.92	1.82	2.733 (4)	173

Symmetry codes: (i) 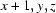; (ii) 
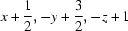
.

**Fig. 1.**
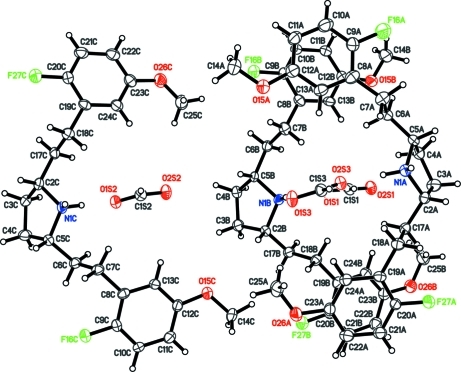
A view of the asymmetric unit with the atom numbering scheme. Displacement ellipsoids are drawn at the 50% probability level.

## supplementary crystallographic information

### Comment

In endeavoring to develop lobelane analogs with high affinity for dihydrotetrabenazine binding sites on VMAT2 and as inhibiters of [^3^H]-DA uptake into cystolic vesicles (Zheng *et al.* (2005)), we have undertaken the design, synthesis and structural analysis of a series of 2,5-disubstitued phenethylpyrrolidine analogs. The primary goal of the X-ray analysis of the title compound was to confirm the stereochemistry of the substituted phenethyl groups in the molecule, and to obtain detailed information on the structural conformation of the molecule that may be useful in structure-activity relationship (SAR) analysis. The title compound is prepared by utilizing a previously reported procedure (Vartak *et al.* 2009). The X-ray studies revealed that the crystal lattice has three independent (2*R*,5*S*)-2,5-bis(2-fluoro-5-methoxyphenethyl) pyrrolidinium formate molecules. The central pyrrolidinium ring is not planar and the 2,5-disubstituted phenylethyl groups are in equatorial positions. The angle between the exact plane defined by C6, C7, C8 and by the mean plane passing closest to the atoms of the pyrrolidinium ring (N1, C2, C3, C4, C5) for molecule A is: 51.8 (3)° and 48.7 (3)°; molecule B is: 48.4 (3)° and 47.8 (3)° and for molecule C is: 48.5 (3)° and 44.8 (3)°. The dihedral angles between C5,C6, C7 plane to C8, C9, C13 planes and C2, C17, C18 plane to C19, C20, C24 plane for ion A is 86.26 (4)° and 73.06 (4)°; for ion B is: 81.87 (4)° and 74.37 (4)° and for ion C is: 72.58 (4)° and 84.89 (4)°. The molecules are linked into dimeric chains by a series of N—H···O hydrogen bonds. Significant intermolecular hydrogen-bonding interactions are found between pyrrolidinium N(1)—H(1)···O (formate anion) and N(1)—H(2)···O (formate anion).

### Experimental

The title compound was prepared by the reaction of (3*S*,5*R*, 7a*R*)-5-(benzotriazol-1-yl)-3-phenyl[2,1-*b*]oxazolopyrrolidine with 2-fluoro 5-methoxyphenethyl magnesium bromide in tetrahydrofuran to afford a mixture of crude 2*R*,5*S*- and 2*R*,5*R*-di-2-fluoro-5-methoxyethylpyrrolidine diastereomers, which are separated by silica gel column chromatography. The obtained 2*R*,5*R* compound was hydrogenolyzed by catalytic-transfer hydrogenation with palladium hydroxide-over-carbon, employing ammonium formate as the hydrogen source in refluxing methanol. These conditions afforded quantitative conversion to the product, (2*R*,5*S*)-2,5-bis-(2-fluoro-5-methoxyphenethyl)pyrrolidine within 30 min. The formate salt is prepared by treatment with formic acid in methylene chloride and recrystallization from diethyl ether. The crystals obtained from the solution of diethyl ether are suitable for X-ray diffraction studies. ^1^H NMR (CDCl_3_): δ 1.60–1.75 (*m*, 2H), 1.80–1.95 (*m*, 2H), 1.95–2.20 (*m*, 4H), 2.52–2.70 (*m*, 4H), 3.28 (*t*, *J*=6.0 Hz, 2H), 3.68 (*s*, 6H), 6.55–6.70 (*m*, 4H), 6.83 (*t*, *J* =9.3 Hz, 2H), 8.58 (*s*, 1H), 9.20 (*bs*, 2H) *p.p.m.*; ^13^C NMR (DMSO d_6_): δ 26.78, 29.68, 33.74, 55.88, 59.23, 112.69, 115.42, 115.59, 128.28, 128.51, 155.66, 168.47 *p.p.m.*.

### Refinement

H atoms were found in difference Fourier maps and subsequently placed in idealized positions with constrained distances of 0.98 Å (RCH_3_), 0.99 Å (*R*_2_CH_2_), 1.00 Å (*R*_3_CH), 0.95 Å (C_Ar_H), 0.92 Å (N—H), and with *U*_iso_(H) values set to either 1.2*U*_eq_ or 1.5*U*_eq_ (RCH_3_) of the attached atom. Since this structure was refined using data from a Mo Kα anode, there is effectively no anomalous signal, and therefore no way to obtain a meaningful Flack parameter. For this reason, the 6574 Friedel pairs were merged prior to the final cycles of refinement.

### Crystal data

**Table:**

C_22_H_28_F_2_NO_2_^+^·CHO_2_^−^	*F*(000) = 2688
*M**_r_* = 421.47	*D*_x_ = 1.312 Mg m^−^^3^
Orthorhombic, *P*2_1_2_1_2_1_	Mo *K*α radiation, λ = 0.71073 Å
Hall symbol: P 2ac 2ab	Cell parameters from 8196 reflections
*a* = 7.8338 (1) Å	θ = 1.0–27.5°
*b* = 27.8759 (3) Å	µ = 0.10 mm^−^^1^
*c* = 29.3202 (3) Å	*T* = 90 K
*V* = 6402.78 (13) Å^3^	Plate, colourless
*Z* = 12	0.28 × 0.18 × 0.06 mm

### Data collection

**Table:**

Nonius KappaCCD diffractometer	8166 independent reflections
Radiation source: fine-focus sealed tube	5100 reflections with *I* > 2σ(*I*)
graphite	*R*_int_ = 0.051
Detector resolution: 9.1 pixels mm^-1^	θ_max_ = 27.5°, θ_min_ = 1.0°
ω scans at fixed χ = 55°	*h* = −10→10
Absorption correction: multi-scan (*SCALEPACK*; Otwinowski & Minor, 1997)	*k* = −36→36
*T*_min_ = 0.973, *T*_max_ = 0.994	*l* = −37→38
80877 measured reflections	

### Refinement

**Table:**

Refinement on *F*^2^	Primary atom site location: structure-invariant direct methods
Least-squares matrix: full	Secondary atom site location: difference Fourier map
*R*[*F*^2^ > 2σ(*F*^2^)] = 0.056	Hydrogen site location: inferred from neighbouring sites
*wR*(*F*^2^) = 0.155	H-atom parameters constrained
*S* = 1.00	*w* = 1/[σ^2^(*F*_o_^2^) + (0.0915*P*)^2^] where *P* = (*F*_o_^2^ + 2*F*_c_^2^)/3
8166 reflections	(Δ/σ)_max_ = 0.001
817 parameters	Δρ_max_ = 0.34 e Å^−^^3^
0 restraints	Δρ_min_ = −0.28 e Å^−^^3^

### Special details

**Table:**

Geometry. All e.s.d.'s (except the e.s.d. in the dihedral angle between two l.s. planes) are estimated using the full covariance matrix. The cell e.s.d.'s are taken into account individually in the estimation of e.s.d.'s in distances, angles and torsion angles; correlations between e.s.d.'s in cell parameters are only used when they are defined by crystal symmetry. An approximate (isotropic) treatment of cell e.s.d.'s is used for estimating e.s.d.'s involving l.s. planes.
Refinement. Refinement of *F*^2^ against ALL reflections. The weighted *R*-factor *wR* and goodness of fit *S* are based on *F*^2^, conventional *R*-factors *R* are based on *F*, with *F* set to zero for negative *F*^2^. The threshold expression of *F*^2^ > 2σ(*F*^2^) is used only for calculating *R*-factors(gt) *etc*. and is not relevant to the choice of reflections for refinement. *R*-factors based on *F*^2^ are statistically about twice as large as those based on *F*, and *R*- factors based on ALL data will be even larger.

### Fractional atomic coordinates and isotropic or equivalent isotropic displacement parameters (Å^2^)

**Table:**

	*x*	*y*	*z*	*U*_iso_*/*U*_eq_	
N1A	0.7000 (4)	0.33489 (10)	0.52187 (9)	0.0234 (7)	
H1A1	0.6523	0.3650	0.5228	0.028*	
H1A2	0.8169	0.3381	0.5218	0.028*	
C2A	0.6434 (5)	0.30603 (12)	0.56265 (11)	0.0243 (8)	
H2A	0.7412	0.2857	0.5731	0.029*	
C3A	0.5015 (6)	0.27303 (13)	0.54478 (12)	0.0324 (10)	
H3A1	0.5356	0.2390	0.5477	0.039*	
H3A2	0.3949	0.2781	0.5623	0.039*	
C4A	0.4750 (5)	0.28602 (12)	0.49485 (12)	0.0248 (9)	
H4A1	0.4500	0.2570	0.4766	0.030*	
H4A2	0.3793	0.3089	0.4914	0.030*	
C5A	0.6423 (5)	0.30877 (12)	0.47979 (11)	0.0217 (8)	
H5A	0.7262	0.2828	0.4727	0.026*	
C6A	0.6285 (5)	0.34214 (12)	0.43896 (11)	0.0243 (8)	
H6A1	0.5540	0.3694	0.4473	0.029*	
H6A2	0.5722	0.3246	0.4137	0.029*	
C7A	0.7984 (6)	0.36200 (13)	0.42158 (12)	0.0331 (10)	
H7A1	0.8629	0.3760	0.4474	0.040*	
H7A2	0.8673	0.3355	0.4086	0.040*	
C8A	0.7702 (5)	0.39970 (14)	0.38574 (12)	0.0302 (9)	
C9A	0.7528 (6)	0.38972 (13)	0.33965 (14)	0.0366 (11)	
C10A	0.7247 (6)	0.42351 (14)	0.30744 (13)	0.0431 (12)	
H10A	0.7149	0.4145	0.2763	0.052*	
C11A	0.7103 (6)	0.47136 (14)	0.32002 (13)	0.0350 (10)	
H11A	0.6901	0.4955	0.2978	0.042*	
C12A	0.7260 (5)	0.48291 (13)	0.36570 (12)	0.0313 (9)	
C13A	0.7538 (5)	0.44768 (13)	0.39795 (12)	0.0287 (9)	
H13A	0.7619	0.4564	0.4292	0.034*	
C14A	0.7149 (8)	0.56681 (13)	0.35088 (14)	0.0605 (16)	
H14A	0.6098	0.5648	0.3328	0.091*	
H14B	0.8139	0.5642	0.3306	0.091*	
H14C	0.7185	0.5976	0.3669	0.091*	
O15A	0.7182 (4)	0.52938 (9)	0.38272 (8)	0.0395 (8)	
F16A	0.7685 (4)	0.34235 (8)	0.32668 (8)	0.0555 (8)	
C17A	0.5908 (5)	0.33851 (12)	0.60142 (11)	0.0225 (8)	
H17A	0.5390	0.3188	0.6258	0.027*	
H17B	0.5023	0.3610	0.5903	0.027*	
C18A	0.7380 (5)	0.36745 (13)	0.62166 (12)	0.0260 (9)	
H18A	0.8298	0.3452	0.6313	0.031*	
H18B	0.7852	0.3889	0.5979	0.031*	
C19A	0.6829 (5)	0.39723 (12)	0.66218 (11)	0.0214 (8)	
C20A	0.6399 (5)	0.37604 (12)	0.70328 (12)	0.0235 (8)	
C21A	0.5787 (5)	0.40074 (13)	0.74026 (13)	0.0287 (9)	
H21A	0.5494	0.3843	0.7676	0.034*	
C22A	0.5604 (5)	0.44993 (13)	0.73709 (12)	0.0294 (9)	
H22A	0.5186	0.4678	0.7623	0.035*	
C23A	0.6033 (5)	0.47283 (12)	0.69689 (12)	0.0261 (9)	
C24A	0.6640 (5)	0.44693 (12)	0.65964 (12)	0.0240 (9)	
H24A	0.6929	0.4633	0.6323	0.029*	
C25A	0.6306 (6)	0.54783 (13)	0.65779 (14)	0.0394 (11)	
H25A	0.5748	0.5350	0.6305	0.059*	
H25B	0.7547	0.5449	0.6546	0.059*	
H25C	0.6002	0.5817	0.6615	0.059*	
O26A	0.5760 (4)	0.52174 (8)	0.69656 (9)	0.0352 (7)	
F27A	0.6578 (3)	0.32703 (7)	0.70685 (7)	0.0347 (6)	
N1B	0.1995 (4)	0.49637 (10)	0.53019 (9)	0.0211 (7)	
H1B1	0.3163	0.4928	0.5303	0.025*	
H1B2	0.1506	0.4664	0.5283	0.025*	
C2B	0.1418 (5)	0.52129 (12)	0.57307 (11)	0.0231 (8)	
H2B	0.2386	0.5409	0.5853	0.028*	
C3B	−0.0018 (5)	0.55540 (13)	0.55755 (12)	0.0313 (10)	
H3B1	−0.1097	0.5475	0.5735	0.038*	
H3B2	0.0286	0.5891	0.5646	0.038*	
C4B	−0.0223 (5)	0.54870 (12)	0.50654 (12)	0.0258 (9)	
H4B1	−0.0426	0.5799	0.4913	0.031*	
H4B2	−0.1192	0.5270	0.4998	0.031*	
C5B	0.1452 (5)	0.52669 (11)	0.49051 (11)	0.0205 (8)	
H5B	0.2307	0.5529	0.4860	0.025*	
C6B	0.1324 (5)	0.49765 (12)	0.44689 (11)	0.0231 (8)	
H6B1	0.0832	0.5183	0.4228	0.028*	
H6B2	0.0518	0.4708	0.4521	0.028*	
C7B	0.3001 (5)	0.47696 (13)	0.42935 (12)	0.0287 (9)	
H7B1	0.3770	0.5034	0.4201	0.034*	
H7B2	0.3568	0.4587	0.4540	0.034*	
C8B	0.2681 (5)	0.44441 (13)	0.38917 (12)	0.0263 (9)	
C9B	0.2464 (6)	0.46143 (13)	0.34513 (13)	0.0357 (11)	
C10B	0.2084 (7)	0.43254 (13)	0.30869 (13)	0.0438 (13)	
H10B	0.1926	0.4459	0.2792	0.053*	
C11B	0.1932 (6)	0.38373 (13)	0.31543 (12)	0.0341 (10)	
H11B	0.1688	0.3631	0.2905	0.041*	
C12B	0.2139 (5)	0.36502 (12)	0.35885 (12)	0.0277 (9)	
C13B	0.2498 (5)	0.39520 (13)	0.39497 (11)	0.0267 (9)	
H13B	0.2624	0.3819	0.4246	0.032*	
C14B	0.1873 (7)	0.28431 (13)	0.33248 (14)	0.0503 (14)	
H14D	0.2829	0.2890	0.3114	0.075*	
H14E	0.0795	0.2902	0.3165	0.075*	
H14F	0.1889	0.2513	0.3440	0.075*	
O15B	0.2027 (4)	0.31685 (8)	0.36954 (8)	0.0364 (7)	
F16B	0.2627 (4)	0.50973 (7)	0.33811 (7)	0.0570 (9)	
C17B	0.0877 (5)	0.48538 (12)	0.60918 (11)	0.0250 (9)	
H17C	−0.0010	0.4641	0.5962	0.030*	
H17D	0.0362	0.5029	0.6351	0.030*	
C18B	0.2359 (5)	0.45452 (12)	0.62692 (11)	0.0238 (8)	
H18C	0.2832	0.4355	0.6014	0.029*	
H18D	0.3276	0.4758	0.6384	0.029*	
C19B	0.1814 (5)	0.42096 (12)	0.66466 (12)	0.0241 (9)	
C20B	0.1457 (5)	0.43818 (12)	0.70796 (12)	0.0276 (9)	
C21B	0.0951 (5)	0.40914 (13)	0.74316 (13)	0.0323 (10)	
H21B	0.0743	0.4222	0.7726	0.039*	
C22B	0.0748 (5)	0.36083 (13)	0.73535 (13)	0.0316 (10)	
H22B	0.0382	0.3403	0.7593	0.038*	
C23B	0.1076 (5)	0.34209 (12)	0.69258 (12)	0.0265 (9)	
C24B	0.1641 (5)	0.37192 (12)	0.65751 (13)	0.0250 (9)	
H24B	0.1909	0.3586	0.6285	0.030*	
C25B	0.1377 (6)	0.27090 (13)	0.64725 (13)	0.0392 (11)	
H25D	0.2593	0.2777	0.6428	0.059*	
H25E	0.0727	0.2832	0.6212	0.059*	
H25F	0.1207	0.2362	0.6498	0.059*	
O26B	0.0807 (4)	0.29337 (8)	0.68753 (8)	0.0342 (7)	
F27B	0.1639 (3)	0.48627 (7)	0.71591 (7)	0.0386 (6)	
N1C	0.2065 (4)	0.83060 (9)	0.49995 (9)	0.0217 (7)	
H1C1	0.3232	0.8270	0.4986	0.026*	
H1C2	0.1574	0.8006	0.4993	0.026*	
C2C	0.1453 (5)	0.85987 (11)	0.46041 (11)	0.0226 (8)	
H2C	0.2294	0.8860	0.4541	0.027*	
C3C	−0.0201 (5)	0.88190 (12)	0.47814 (11)	0.0244 (9)	
H3C1	−0.0443	0.9126	0.4625	0.029*	
H3C2	−0.1173	0.8598	0.4732	0.029*	
C4C	0.0097 (5)	0.88998 (13)	0.52889 (12)	0.0310 (9)	
H4C1	−0.0949	0.8823	0.5464	0.037*	
H4C2	0.0406	0.9239	0.5347	0.037*	
C5C	0.1567 (5)	0.85652 (11)	0.54309 (11)	0.0226 (8)	
H5C	0.2553	0.8767	0.5532	0.027*	
C6C	0.1158 (5)	0.82101 (12)	0.58041 (11)	0.0247 (8)	
H6C1	0.0252	0.7991	0.5695	0.030*	
H6C2	0.0705	0.8386	0.6071	0.030*	
C7C	0.2707 (5)	0.79116 (12)	0.59547 (11)	0.0243 (9)	
H7C1	0.3130	0.7724	0.5691	0.029*	
H7C2	0.3631	0.8131	0.6052	0.029*	
C8C	0.2290 (5)	0.75704 (13)	0.63434 (11)	0.0230 (8)	
C9C	0.1995 (5)	0.77341 (12)	0.67784 (12)	0.0258 (9)	
C10C	0.1592 (5)	0.74395 (12)	0.71369 (12)	0.0272 (9)	
H10C	0.1365	0.7570	0.7430	0.033*	
C11C	0.1521 (5)	0.69478 (13)	0.70668 (12)	0.0290 (9)	
H11C	0.1272	0.6736	0.7312	0.035*	
C12C	0.1824 (5)	0.67695 (12)	0.66286 (12)	0.0259 (9)	
C13C	0.2188 (5)	0.70811 (12)	0.62752 (12)	0.0238 (9)	
H13C	0.2372	0.6956	0.5978	0.029*	
C14C	0.1721 (7)	0.59550 (12)	0.68860 (14)	0.0495 (14)	
H14G	0.0598	0.5975	0.7032	0.074*	
H14H	0.2610	0.6032	0.7109	0.074*	
H14I	0.1901	0.5629	0.6770	0.074*	
O15C	0.1799 (4)	0.62879 (8)	0.65165 (8)	0.0379 (8)	
F16C	0.2088 (3)	0.82192 (7)	0.68536 (7)	0.0363 (6)	
C17C	0.1235 (5)	0.82966 (12)	0.41795 (11)	0.0235 (8)	
H17E	0.0706	0.8498	0.3940	0.028*	
H17F	0.0430	0.8033	0.4249	0.028*	
C18C	0.2875 (5)	0.80786 (12)	0.39878 (11)	0.0253 (9)	
H18E	0.3666	0.8338	0.3896	0.030*	
H18F	0.3443	0.7884	0.4226	0.030*	
C19C	0.2472 (5)	0.77652 (12)	0.35794 (11)	0.0240 (9)	
C20C	0.2247 (5)	0.79575 (12)	0.31494 (12)	0.0278 (9)	
C21C	0.1729 (5)	0.76943 (14)	0.27767 (12)	0.0333 (10)	
H21C	0.1587	0.7842	0.2487	0.040*	
C22C	0.1419 (5)	0.72082 (13)	0.28340 (12)	0.0312 (10)	
H22C	0.1077	0.7018	0.2581	0.037*	
C23C	0.1607 (5)	0.69997 (13)	0.32604 (13)	0.0289 (9)	
C24C	0.2143 (5)	0.72757 (13)	0.36283 (12)	0.0267 (9)	
H24C	0.2288	0.7129	0.3918	0.032*	
C25C	0.1776 (6)	0.62580 (13)	0.36764 (13)	0.0402 (11)	
H25G	0.1243	0.6393	0.3950	0.060*	
H25H	0.3021	0.6281	0.3704	0.060*	
H25I	0.1445	0.5921	0.3645	0.060*	
O26C	0.1229 (4)	0.65175 (9)	0.32878 (9)	0.0368 (7)	
F27C	0.2552 (3)	0.84398 (7)	0.30887 (7)	0.0399 (6)	
C1S1	0.1081 (5)	0.37264 (12)	0.52746 (12)	0.0259 (9)	
H1S1	0.2286	0.3755	0.5296	0.031*	
O1S1	0.0262 (4)	0.41060 (9)	0.52353 (9)	0.0310 (7)	
O2S1	0.0481 (4)	0.33102 (9)	0.52884 (9)	0.0308 (7)	
C1S2	0.1155 (5)	0.70727 (12)	0.49452 (11)	0.0255 (8)	
H1S2	0.2358	0.7101	0.4915	0.031*	
O1S2	0.0346 (4)	0.74531 (9)	0.49840 (9)	0.0278 (6)	
O2S2	0.0557 (4)	0.66551 (9)	0.49421 (9)	0.0322 (7)	
C1S3	0.6079 (5)	0.45870 (12)	0.53183 (12)	0.0261 (9)	
H1S3	0.7286	0.4561	0.5338	0.031*	
O1S3	0.5466 (3)	0.49960 (8)	0.53746 (9)	0.0320 (7)	
O2S3	0.5280 (3)	0.42086 (8)	0.52393 (8)	0.0271 (6)	

### Atomic displacement parameters (Å^2^)

**Table:**

	*U*^11^	*U*^22^	*U*^33^	*U*^12^	*U*^13^	*U*^23^
N1A	0.0222 (18)	0.0227 (16)	0.0252 (16)	−0.0007 (14)	0.0016 (14)	0.0002 (13)
C2A	0.029 (2)	0.0198 (18)	0.0240 (18)	−0.0024 (17)	0.0014 (18)	0.0017 (15)
C3A	0.038 (3)	0.031 (2)	0.028 (2)	−0.010 (2)	0.002 (2)	0.0001 (17)
C4A	0.024 (2)	0.0209 (19)	0.030 (2)	−0.0049 (17)	−0.0005 (18)	−0.0030 (16)
C5A	0.022 (2)	0.0218 (18)	0.0217 (18)	0.0015 (17)	0.0014 (17)	−0.0037 (14)
C6A	0.029 (2)	0.0227 (19)	0.0213 (18)	0.0025 (18)	−0.0004 (17)	−0.0024 (15)
C7A	0.034 (2)	0.036 (2)	0.030 (2)	−0.002 (2)	0.000 (2)	0.0054 (18)
C8A	0.024 (2)	0.038 (2)	0.029 (2)	−0.0033 (19)	0.0007 (18)	0.0078 (17)
C9A	0.050 (3)	0.025 (2)	0.035 (2)	−0.001 (2)	0.007 (2)	0.0001 (18)
C10A	0.066 (3)	0.038 (2)	0.026 (2)	−0.005 (2)	−0.004 (2)	0.0023 (19)
C11A	0.044 (3)	0.033 (2)	0.028 (2)	0.000 (2)	−0.004 (2)	0.0053 (17)
C12A	0.033 (2)	0.033 (2)	0.028 (2)	−0.001 (2)	0.000 (2)	0.0018 (17)
C13A	0.030 (2)	0.032 (2)	0.0241 (18)	−0.0048 (19)	0.0020 (19)	0.0030 (16)
C14A	0.109 (5)	0.031 (2)	0.041 (2)	0.010 (3)	0.004 (3)	0.007 (2)
O15A	0.058 (2)	0.0286 (15)	0.0318 (14)	0.0059 (15)	0.0005 (15)	0.0012 (12)
F16A	0.094 (2)	0.0329 (13)	0.0399 (13)	−0.0057 (15)	0.0023 (15)	−0.0009 (11)
C17A	0.023 (2)	0.0212 (18)	0.0236 (18)	−0.0026 (17)	0.0011 (17)	0.0020 (15)
C18A	0.025 (2)	0.029 (2)	0.0246 (19)	−0.0029 (18)	0.0023 (18)	−0.0009 (15)
C19A	0.018 (2)	0.028 (2)	0.0191 (17)	0.0001 (17)	0.0002 (16)	−0.0026 (15)
C20A	0.029 (2)	0.0186 (19)	0.0225 (18)	−0.0015 (17)	−0.0060 (18)	−0.0019 (15)
C21A	0.032 (2)	0.031 (2)	0.0229 (19)	−0.0016 (19)	−0.0006 (18)	−0.0010 (17)
C22A	0.033 (2)	0.031 (2)	0.0237 (19)	−0.0054 (19)	0.0015 (18)	−0.0030 (17)
C23A	0.029 (2)	0.0209 (19)	0.029 (2)	0.0007 (18)	−0.0023 (19)	0.0012 (16)
C24A	0.026 (2)	0.0232 (19)	0.0227 (18)	−0.0073 (17)	0.0009 (17)	0.0015 (15)
C25A	0.049 (3)	0.025 (2)	0.044 (2)	−0.007 (2)	0.005 (2)	0.0036 (18)
O26A	0.0481 (19)	0.0240 (14)	0.0336 (15)	0.0007 (14)	0.0082 (15)	0.0004 (12)
F27A	0.0526 (16)	0.0231 (11)	0.0286 (11)	0.0015 (11)	0.0036 (11)	0.0010 (9)
N1B	0.0200 (17)	0.0221 (15)	0.0211 (15)	0.0036 (14)	0.0007 (14)	0.0021 (12)
C2B	0.026 (2)	0.0204 (18)	0.0224 (18)	0.0033 (17)	0.0011 (17)	−0.0024 (14)
C3B	0.035 (2)	0.031 (2)	0.028 (2)	0.012 (2)	0.0026 (19)	0.0011 (17)
C4B	0.027 (2)	0.0199 (19)	0.030 (2)	0.0001 (18)	−0.0009 (18)	−0.0011 (16)
C5B	0.025 (2)	0.0152 (17)	0.0207 (17)	0.0017 (16)	0.0020 (16)	0.0026 (14)
C6B	0.024 (2)	0.0216 (18)	0.0237 (19)	0.0015 (17)	−0.0014 (17)	0.0011 (15)
C7B	0.027 (2)	0.027 (2)	0.032 (2)	0.0024 (19)	−0.0014 (19)	−0.0021 (16)
C8B	0.024 (2)	0.026 (2)	0.029 (2)	0.0036 (18)	0.0041 (18)	−0.0041 (16)
C9B	0.059 (3)	0.020 (2)	0.028 (2)	0.001 (2)	0.007 (2)	0.0002 (16)
C10B	0.082 (4)	0.027 (2)	0.023 (2)	0.009 (2)	0.010 (2)	−0.0003 (17)
C11B	0.052 (3)	0.029 (2)	0.0221 (19)	0.005 (2)	0.001 (2)	−0.0040 (16)
C12B	0.031 (2)	0.024 (2)	0.028 (2)	0.0018 (18)	0.0002 (19)	−0.0023 (16)
C13B	0.031 (2)	0.031 (2)	0.0179 (17)	0.0063 (19)	0.0037 (17)	−0.0021 (15)
C14B	0.085 (4)	0.029 (2)	0.036 (2)	−0.004 (3)	0.002 (3)	−0.0073 (19)
O15B	0.055 (2)	0.0270 (14)	0.0273 (13)	−0.0026 (15)	0.0022 (15)	−0.0028 (11)
F16B	0.112 (3)	0.0245 (13)	0.0344 (13)	0.0018 (15)	0.0140 (16)	0.0006 (10)
C17B	0.027 (2)	0.027 (2)	0.0213 (18)	0.0017 (18)	0.0002 (17)	0.0013 (15)
C18B	0.023 (2)	0.0261 (19)	0.0223 (17)	−0.0002 (18)	0.0023 (17)	0.0041 (15)
C19B	0.021 (2)	0.026 (2)	0.0258 (19)	0.0012 (17)	0.0008 (17)	0.0041 (16)
C20B	0.035 (2)	0.022 (2)	0.026 (2)	−0.0009 (18)	−0.0031 (19)	0.0007 (15)
C21B	0.044 (3)	0.033 (2)	0.0207 (18)	−0.001 (2)	0.002 (2)	−0.0005 (17)
C22B	0.033 (2)	0.033 (2)	0.029 (2)	0.0024 (19)	0.0042 (19)	0.0056 (18)
C23B	0.025 (2)	0.025 (2)	0.030 (2)	−0.0013 (18)	−0.0032 (18)	−0.0007 (16)
C24B	0.023 (2)	0.027 (2)	0.0252 (19)	0.0019 (18)	0.0027 (17)	0.0005 (16)
C25B	0.043 (3)	0.033 (2)	0.042 (2)	0.004 (2)	0.006 (2)	−0.0021 (19)
O26B	0.0428 (19)	0.0256 (14)	0.0342 (15)	−0.0055 (13)	0.0058 (14)	0.0028 (12)
F27B	0.0566 (17)	0.0275 (12)	0.0318 (12)	−0.0022 (12)	0.0004 (12)	0.0016 (10)
N1C	0.0238 (17)	0.0165 (15)	0.0246 (16)	0.0031 (14)	−0.0013 (15)	−0.0002 (12)
C2C	0.024 (2)	0.0186 (18)	0.0256 (18)	−0.0022 (16)	−0.0013 (17)	0.0036 (14)
C3C	0.025 (2)	0.0241 (19)	0.0240 (19)	0.0007 (17)	0.0002 (17)	0.0039 (16)
C4C	0.035 (3)	0.036 (2)	0.0223 (19)	0.009 (2)	0.0032 (19)	−0.0005 (17)
C5C	0.027 (2)	0.0198 (18)	0.0215 (17)	0.0008 (17)	−0.0004 (17)	−0.0016 (14)
C6C	0.026 (2)	0.0267 (19)	0.0211 (18)	0.0005 (18)	0.0027 (17)	0.0021 (15)
C7C	0.025 (2)	0.0274 (19)	0.0206 (18)	−0.0023 (18)	0.0038 (17)	0.0019 (15)
C8C	0.020 (2)	0.029 (2)	0.0200 (18)	0.0039 (17)	−0.0019 (17)	0.0036 (15)
C9C	0.032 (2)	0.0178 (19)	0.028 (2)	−0.0013 (18)	0.0007 (19)	−0.0017 (15)
C10C	0.040 (3)	0.025 (2)	0.0169 (17)	0.0009 (18)	0.0013 (18)	0.0027 (15)
C11C	0.035 (2)	0.027 (2)	0.0247 (19)	−0.0021 (19)	−0.0006 (19)	0.0051 (16)
C12C	0.030 (2)	0.0203 (19)	0.0271 (19)	0.0005 (18)	−0.0062 (18)	0.0028 (16)
C13C	0.029 (2)	0.0240 (19)	0.0188 (17)	0.0051 (18)	−0.0001 (17)	−0.0012 (15)
C14C	0.095 (4)	0.019 (2)	0.034 (2)	−0.004 (2)	−0.005 (3)	0.0069 (17)
O15C	0.064 (2)	0.0209 (14)	0.0286 (14)	0.0002 (15)	−0.0058 (15)	−0.0006 (11)
F16C	0.0566 (17)	0.0216 (11)	0.0306 (12)	−0.0011 (11)	0.0030 (12)	−0.0013 (9)
C17C	0.027 (2)	0.0185 (18)	0.0254 (18)	−0.0025 (17)	−0.0014 (17)	−0.0002 (15)
C18C	0.023 (2)	0.028 (2)	0.0247 (19)	0.0015 (18)	−0.0022 (18)	−0.0017 (16)
C19C	0.021 (2)	0.028 (2)	0.0224 (18)	0.0073 (18)	0.0002 (17)	−0.0055 (15)
C20C	0.033 (2)	0.022 (2)	0.028 (2)	0.0015 (18)	0.0018 (19)	−0.0003 (16)
C21C	0.039 (3)	0.039 (2)	0.0226 (19)	0.004 (2)	−0.0031 (19)	−0.0035 (17)
C22C	0.031 (2)	0.037 (2)	0.025 (2)	0.002 (2)	−0.0046 (19)	−0.0058 (17)
C23C	0.028 (2)	0.028 (2)	0.031 (2)	−0.0010 (19)	−0.0037 (19)	−0.0058 (17)
C24C	0.028 (2)	0.027 (2)	0.0252 (19)	0.0035 (18)	−0.0009 (18)	−0.0033 (16)
C25C	0.053 (3)	0.030 (2)	0.038 (2)	0.005 (2)	−0.009 (2)	−0.0005 (18)
O26C	0.0473 (19)	0.0285 (15)	0.0346 (15)	−0.0012 (15)	−0.0121 (15)	−0.0036 (12)
F27C	0.0573 (18)	0.0298 (12)	0.0326 (12)	−0.0010 (12)	−0.0016 (13)	−0.0003 (10)
C1S1	0.026 (2)	0.023 (2)	0.0284 (19)	0.0001 (18)	−0.0008 (18)	−0.0039 (16)
O1S1	0.0310 (17)	0.0246 (14)	0.0375 (15)	0.0011 (13)	0.0007 (14)	0.0016 (12)
O2S1	0.0284 (17)	0.0239 (14)	0.0400 (15)	−0.0026 (13)	0.0001 (13)	−0.0013 (12)
C1S2	0.022 (2)	0.027 (2)	0.0274 (19)	−0.0033 (18)	−0.0028 (18)	0.0004 (16)
O1S2	0.0289 (15)	0.0225 (13)	0.0320 (13)	0.0015 (13)	−0.0015 (13)	−0.0012 (11)
O2S2	0.0305 (17)	0.0215 (14)	0.0448 (16)	−0.0047 (12)	0.0008 (14)	0.0013 (12)
C1S3	0.024 (2)	0.027 (2)	0.028 (2)	0.0031 (18)	−0.0017 (18)	−0.0015 (17)
O1S3	0.0269 (16)	0.0224 (14)	0.0466 (17)	0.0034 (13)	−0.0005 (14)	0.0025 (13)
O2S3	0.0262 (16)	0.0221 (13)	0.0330 (14)	0.0016 (12)	0.0001 (13)	−0.0016 (12)

### Geometric parameters (Å, °)

**Table:**

N1A—C5A	1.502 (4)	C14B—H14D	0.9800
N1A—C2A	1.508 (4)	C14B—H14E	0.9800
N1A—H1A1	0.9200	C14B—H14F	0.9800
N1A—H1A2	0.9200	C17B—C18B	1.536 (5)
C2A—C17A	1.511 (5)	C17B—H17C	0.9900
C2A—C3A	1.535 (5)	C17B—H17D	0.9900
C2A—H2A	1.0000	C18B—C19B	1.511 (5)
C3A—C4A	1.522 (5)	C18B—H18C	0.9900
C3A—H3A1	0.9900	C18B—H18D	0.9900
C3A—H3A2	0.9900	C19B—C20B	1.386 (5)
C4A—C5A	1.522 (5)	C19B—C24B	1.390 (5)
C4A—H4A1	0.9900	C20B—F27B	1.368 (4)
C4A—H4A2	0.9900	C20B—C21B	1.370 (5)
C5A—C6A	1.520 (5)	C21B—C22B	1.375 (5)
C5A—H5A	1.0000	C21B—H21B	0.9500
C6A—C7A	1.529 (5)	C22B—C23B	1.383 (5)
C6A—H6A1	0.9900	C22B—H22B	0.9500
C6A—H6A2	0.9900	C23B—O26B	1.382 (4)
C7A—C8A	1.503 (5)	C23B—C24B	1.395 (5)
C7A—H7A1	0.9900	C24B—H24B	0.9500
C7A—H7A2	0.9900	C25B—O26B	1.409 (4)
C8A—C9A	1.386 (5)	C25B—H25D	0.9800
C8A—C13A	1.391 (5)	C25B—H25E	0.9800
C9A—C10A	1.352 (5)	C25B—H25F	0.9800
C9A—F16A	1.380 (4)	N1C—C2C	1.496 (4)
C10A—C11A	1.388 (5)	N1C—C5C	1.508 (4)
C10A—H10A	0.9500	N1C—H1C1	0.9200
C11A—C12A	1.383 (5)	N1C—H1C2	0.9200
C11A—H11A	0.9500	C2C—C17C	1.513 (4)
C12A—C13A	1.380 (5)	C2C—C3C	1.525 (5)
C12A—O15A	1.389 (4)	C2C—H2C	1.0000
C13A—H13A	0.9500	C3C—C4C	1.523 (5)
C14A—O15A	1.400 (4)	C3C—H3C1	0.9900
C14A—H14A	0.9800	C3C—H3C2	0.9900
C14A—H14B	0.9800	C4C—C5C	1.539 (5)
C14A—H14C	0.9800	C4C—H4C1	0.9900
C17A—C18A	1.528 (5)	C4C—H4C2	0.9900
C17A—H17A	0.9900	C5C—C6C	1.510 (4)
C17A—H17B	0.9900	C5C—H5C	1.0000
C18A—C19A	1.512 (5)	C6C—C7C	1.536 (5)
C18A—H18A	0.9900	C6C—H6C1	0.9900
C18A—H18B	0.9900	C6C—H6C2	0.9900
C19A—C20A	1.384 (5)	C7C—C8C	1.520 (5)
C19A—C24A	1.395 (5)	C7C—H7C1	0.9900
C20A—C21A	1.371 (5)	C7C—H7C2	0.9900
C20A—F27A	1.377 (4)	C8C—C9C	1.374 (5)
C21A—C22A	1.382 (5)	C8C—C13C	1.381 (5)
C21A—H21A	0.9500	C9C—C10C	1.371 (5)
C22A—C23A	1.382 (5)	C9C—F16C	1.372 (4)
C22A—H22A	0.9500	C10C—C11C	1.387 (5)
C23A—O26A	1.380 (4)	C10C—H10C	0.9500
C23A—C24A	1.393 (5)	C11C—C12C	1.398 (5)
C24A—H24A	0.9500	C11C—H11C	0.9500
C25A—O26A	1.416 (4)	C12C—O15C	1.382 (4)
C25A—H25A	0.9800	C12C—C13C	1.382 (5)
C25A—H25B	0.9800	C13C—H13C	0.9500
C25A—H25C	0.9800	C14C—O15C	1.428 (4)
N1B—C5B	1.500 (4)	C14C—H14G	0.9800
N1B—C2B	1.506 (4)	C14C—H14H	0.9800
N1B—H1B1	0.9200	C14C—H14I	0.9800
N1B—H1B2	0.9200	C17C—C18C	1.528 (5)
C2B—C17B	1.517 (4)	C17C—H17E	0.9900
C2B—C3B	1.542 (5)	C17C—H17F	0.9900
C2B—H2B	1.0000	C18C—C19C	1.516 (5)
C3B—C4B	1.516 (5)	C18C—H18E	0.9900
C3B—H3B1	0.9900	C18C—H18F	0.9900
C3B—H3B2	0.9900	C19C—C20C	1.381 (5)
C4B—C5B	1.522 (5)	C19C—C24C	1.396 (5)
C4B—H4B1	0.9900	C20C—C21C	1.378 (5)
C4B—H4B2	0.9900	C20C—F27C	1.377 (4)
C5B—C6B	1.517 (4)	C21C—C22C	1.387 (5)
C5B—H5B	1.0000	C21C—H21C	0.9500
C6B—C7B	1.524 (5)	C22C—C23C	1.387 (5)
C6B—H6B1	0.9900	C22C—H22C	0.9500
C6B—H6B2	0.9900	C23C—O26C	1.379 (4)
C7B—C8B	1.508 (5)	C23C—C24C	1.390 (5)
C7B—H7B1	0.9900	C24C—H24C	0.9500
C7B—H7B2	0.9900	C25C—O26C	1.416 (4)
C8B—C9B	1.386 (5)	C25C—H25G	0.9800
C8B—C13B	1.390 (5)	C25C—H25H	0.9800
C9B—F16B	1.368 (4)	C25C—H25I	0.9800
C9B—C10B	1.371 (5)	C1S1—O1S1	1.243 (4)
C10B—C11B	1.380 (5)	C1S1—O2S1	1.252 (4)
C10B—H10B	0.9500	C1S1—H1S1	0.9500
C11B—C12B	1.385 (5)	C1S2—O1S2	1.240 (4)
C11B—H11B	0.9500	C1S2—O2S2	1.255 (4)
C12B—C13B	1.382 (5)	C1S2—H1S2	0.9500
C12B—O15B	1.381 (4)	C1S3—O2S3	1.249 (4)
C13B—H13B	0.9500	C1S3—O1S3	1.248 (4)
C14B—O15B	1.421 (4)	C1S3—H1S3	0.9500
			
C5A—N1A—C2A	107.7 (2)	C12B—C13B—C8B	121.9 (3)
C5A—N1A—H1A1	110.2	C12B—C13B—H13B	119.1
C2A—N1A—H1A1	110.2	C8B—C13B—H13B	119.1
C5A—N1A—H1A2	110.2	O15B—C14B—H14D	109.5
C2A—N1A—H1A2	110.2	O15B—C14B—H14E	109.5
H1A1—N1A—H1A2	108.5	H14D—C14B—H14E	109.5
N1A—C2A—C17A	110.9 (3)	O15B—C14B—H14F	109.5
N1A—C2A—C3A	105.2 (3)	H14D—C14B—H14F	109.5
C17A—C2A—C3A	114.7 (3)	H14E—C14B—H14F	109.5
N1A—C2A—H2A	108.6	C12B—O15B—C14B	116.9 (3)
C17A—C2A—H2A	108.6	C2B—C17B—C18B	113.3 (3)
C3A—C2A—H2A	108.6	C2B—C17B—H17C	108.9
C4A—C3A—C2A	106.5 (3)	C18B—C17B—H17C	108.9
C4A—C3A—H3A1	110.4	C2B—C17B—H17D	108.9
C2A—C3A—H3A1	110.4	C18B—C17B—H17D	108.9
C4A—C3A—H3A2	110.4	H17C—C17B—H17D	107.7
C2A—C3A—H3A2	110.4	C19B—C18B—C17B	112.4 (3)
H3A1—C3A—H3A2	108.6	C19B—C18B—H18C	109.1
C5A—C4A—C3A	105.1 (3)	C17B—C18B—H18C	109.1
C5A—C4A—H4A1	110.7	C19B—C18B—H18D	109.1
C3A—C4A—H4A1	110.7	C17B—C18B—H18D	109.1
C5A—C4A—H4A2	110.7	H18C—C18B—H18D	107.9
C3A—C4A—H4A2	110.7	C20B—C19B—C24B	117.3 (3)
H4A1—C4A—H4A2	108.8	C20B—C19B—C18B	120.9 (3)
N1A—C5A—C6A	111.8 (3)	C24B—C19B—C18B	121.7 (3)
N1A—C5A—C4A	102.9 (3)	F27B—C20B—C21B	118.7 (3)
C6A—C5A—C4A	115.0 (3)	F27B—C20B—C19B	118.3 (3)
N1A—C5A—H5A	109.0	C21B—C20B—C19B	122.9 (3)
C6A—C5A—H5A	109.0	C20B—C21B—C22B	119.1 (4)
C4A—C5A—H5A	109.0	C20B—C21B—H21B	120.4
C5A—C6A—C7A	115.0 (3)	C22B—C21B—H21B	120.4
C5A—C6A—H6A1	108.5	C21B—C22B—C23B	120.0 (4)
C7A—C6A—H6A1	108.5	C21B—C22B—H22B	120.0
C5A—C6A—H6A2	108.5	C23B—C22B—H22B	120.0
C7A—C6A—H6A2	108.5	O26B—C23B—C22B	116.1 (3)
H6A1—C6A—H6A2	107.5	O26B—C23B—C24B	123.7 (3)
C8A—C7A—C6A	111.0 (3)	C22B—C23B—C24B	120.2 (3)
C8A—C7A—H7A1	109.4	C19B—C24B—C23B	120.4 (3)
C6A—C7A—H7A1	109.4	C19B—C24B—H24B	119.8
C8A—C7A—H7A2	109.4	C23B—C24B—H24B	119.8
C6A—C7A—H7A2	109.4	O26B—C25B—H25D	109.5
H7A1—C7A—H7A2	108.0	O26B—C25B—H25E	109.5
C9A—C8A—C13A	115.8 (3)	H25D—C25B—H25E	109.5
C9A—C8A—C7A	123.8 (4)	O26B—C25B—H25F	109.5
C13A—C8A—C7A	120.4 (3)	H25D—C25B—H25F	109.5
C10A—C9A—F16A	119.3 (4)	H25E—C25B—H25F	109.5
C10A—C9A—C8A	123.9 (4)	C23B—O26B—C25B	118.6 (3)
F16A—C9A—C8A	116.9 (3)	C2C—N1C—C5C	107.8 (2)
C9A—C10A—C11A	119.8 (4)	C2C—N1C—H1C1	110.1
C9A—C10A—H10A	120.1	C5C—N1C—H1C1	110.1
C11A—C10A—H10A	120.1	C2C—N1C—H1C2	110.1
C12A—C11A—C10A	118.3 (4)	C5C—N1C—H1C2	110.1
C12A—C11A—H11A	120.9	H1C1—N1C—H1C2	108.5
C10A—C11A—H11A	120.9	N1C—C2C—C17C	111.7 (3)
C13A—C12A—C11A	120.8 (4)	N1C—C2C—C3C	103.2 (3)
C13A—C12A—O15A	115.1 (3)	C17C—C2C—C3C	114.1 (3)
C11A—C12A—O15A	124.1 (3)	N1C—C2C—H2C	109.2
C12A—C13A—C8A	121.5 (3)	C17C—C2C—H2C	109.2
C12A—C13A—H13A	119.2	C3C—C2C—H2C	109.2
C8A—C13A—H13A	119.2	C4C—C3C—C2C	105.2 (3)
O15A—C14A—H14A	109.5	C4C—C3C—H3C1	110.7
O15A—C14A—H14B	109.5	C2C—C3C—H3C1	110.7
H14A—C14A—H14B	109.5	C4C—C3C—H3C2	110.7
O15A—C14A—H14C	109.5	C2C—C3C—H3C2	110.7
H14A—C14A—H14C	109.5	H3C1—C3C—H3C2	108.8
H14B—C14A—H14C	109.5	C3C—C4C—C5C	106.8 (3)
C12A—O15A—C14A	117.1 (3)	C3C—C4C—H4C1	110.4
C2A—C17A—C18A	113.7 (3)	C5C—C4C—H4C1	110.4
C2A—C17A—H17A	108.8	C3C—C4C—H4C2	110.4
C18A—C17A—H17A	108.8	C5C—C4C—H4C2	110.4
C2A—C17A—H17B	108.8	H4C1—C4C—H4C2	108.6
C18A—C17A—H17B	108.8	N1C—C5C—C6C	110.4 (3)
H17A—C17A—H17B	107.7	N1C—C5C—C4C	104.9 (3)
C19A—C18A—C17A	112.3 (3)	C6C—C5C—C4C	115.8 (3)
C19A—C18A—H18A	109.1	N1C—C5C—H5C	108.5
C17A—C18A—H18A	109.1	C6C—C5C—H5C	108.5
C19A—C18A—H18B	109.1	C4C—C5C—H5C	108.5
C17A—C18A—H18B	109.1	C5C—C6C—C7C	113.3 (3)
H18A—C18A—H18B	107.9	C5C—C6C—H6C1	108.9
C20A—C19A—C24A	116.4 (3)	C7C—C6C—H6C1	108.9
C20A—C19A—C18A	121.3 (3)	C5C—C6C—H6C2	108.9
C24A—C19A—C18A	122.2 (3)	C7C—C6C—H6C2	108.9
C21A—C20A—F27A	118.3 (3)	H6C1—C6C—H6C2	107.7
C21A—C20A—C19A	124.0 (3)	C8C—C7C—C6C	112.7 (3)
F27A—C20A—C19A	117.7 (3)	C8C—C7C—H7C1	109.1
C20A—C21A—C22A	118.8 (4)	C6C—C7C—H7C1	109.1
C20A—C21A—H21A	120.6	C8C—C7C—H7C2	109.1
C22A—C21A—H21A	120.6	C6C—C7C—H7C2	109.1
C23A—C22A—C21A	119.4 (4)	H7C1—C7C—H7C2	107.8
C23A—C22A—H22A	120.3	C9C—C8C—C13C	116.9 (3)
C21A—C22A—H22A	120.3	C9C—C8C—C7C	121.6 (3)
O26A—C23A—C22A	115.1 (3)	C13C—C8C—C7C	121.5 (3)
O26A—C23A—C24A	124.0 (3)	C10C—C9C—F16C	118.6 (3)
C22A—C23A—C24A	120.8 (3)	C10C—C9C—C8C	123.5 (3)
C23A—C24A—C19A	120.6 (3)	F16C—C9C—C8C	117.9 (3)
C23A—C24A—H24A	119.7	C9C—C10C—C11C	119.2 (3)
C19A—C24A—H24A	119.7	C9C—C10C—H10C	120.4
O26A—C25A—H25A	109.5	C11C—C10C—H10C	120.4
O26A—C25A—H25B	109.5	C10C—C11C—C12C	118.7 (3)
H25A—C25A—H25B	109.5	C10C—C11C—H11C	120.6
O26A—C25A—H25C	109.5	C12C—C11C—H11C	120.6
H25A—C25A—H25C	109.5	O15C—C12C—C13C	115.8 (3)
H25B—C25A—H25C	109.5	O15C—C12C—C11C	124.1 (3)
C23A—O26A—C25A	117.8 (3)	C13C—C12C—C11C	120.1 (3)
C5B—N1B—C2B	107.6 (2)	C8C—C13C—C12C	121.6 (3)
C5B—N1B—H1B1	110.2	C8C—C13C—H13C	119.2
C2B—N1B—H1B1	110.2	C12C—C13C—H13C	119.2
C5B—N1B—H1B2	110.2	O15C—C14C—H14G	109.5
C2B—N1B—H1B2	110.2	O15C—C14C—H14H	109.5
H1B1—N1B—H1B2	108.5	H14G—C14C—H14H	109.5
N1B—C2B—C17B	111.2 (3)	O15C—C14C—H14I	109.5
N1B—C2B—C3B	104.9 (3)	H14G—C14C—H14I	109.5
C17B—C2B—C3B	114.1 (3)	H14H—C14C—H14I	109.5
N1B—C2B—H2B	108.8	C12C—O15C—C14C	116.8 (3)
C17B—C2B—H2B	108.8	C2C—C17C—C18C	115.4 (3)
C3B—C2B—H2B	108.8	C2C—C17C—H17E	108.4
C4B—C3B—C2B	107.0 (3)	C18C—C17C—H17E	108.4
C4B—C3B—H3B1	110.3	C2C—C17C—H17F	108.4
C2B—C3B—H3B1	110.3	C18C—C17C—H17F	108.4
C4B—C3B—H3B2	110.3	H17E—C17C—H17F	107.5
C2B—C3B—H3B2	110.3	C19C—C18C—C17C	110.1 (3)
H3B1—C3B—H3B2	108.6	C19C—C18C—H18E	109.6
C3B—C4B—C5B	105.3 (3)	C17C—C18C—H18E	109.6
C3B—C4B—H4B1	110.7	C19C—C18C—H18F	109.6
C5B—C4B—H4B1	110.7	C17C—C18C—H18F	109.6
C3B—C4B—H4B2	110.7	H18E—C18C—H18F	108.1
C5B—C4B—H4B2	110.7	C20C—C19C—C24C	116.7 (3)
H4B1—C4B—H4B2	108.8	C20C—C19C—C18C	121.6 (3)
N1B—C5B—C6B	111.8 (3)	C24C—C19C—C18C	121.4 (3)
N1B—C5B—C4B	103.4 (3)	C21C—C20C—F27C	117.9 (3)
C6B—C5B—C4B	114.8 (3)	C21C—C20C—C19C	123.7 (3)
N1B—C5B—H5B	108.9	F27C—C20C—C19C	118.3 (3)
C6B—C5B—H5B	108.9	C20C—C21C—C22C	118.4 (3)
C4B—C5B—H5B	108.9	C20C—C21C—H21C	120.8
C5B—C6B—C7B	115.4 (3)	C22C—C21C—H21C	120.8
C5B—C6B—H6B1	108.4	C21C—C22C—C23C	120.0 (3)
C7B—C6B—H6B1	108.4	C21C—C22C—H22C	120.0
C5B—C6B—H6B2	108.4	C23C—C22C—H22C	120.0
C7B—C6B—H6B2	108.4	O26C—C23C—C22C	116.0 (3)
H6B1—C6B—H6B2	107.5	O26C—C23C—C24C	124.0 (3)
C8B—C7B—C6B	110.4 (3)	C22C—C23C—C24C	120.0 (3)
C8B—C7B—H7B1	109.6	C23C—C24C—C19C	121.2 (3)
C6B—C7B—H7B1	109.6	C23C—C24C—H24C	119.4
C8B—C7B—H7B2	109.6	C19C—C24C—H24C	119.4
C6B—C7B—H7B2	109.6	O26C—C25C—H25G	109.5
H7B1—C7B—H7B2	108.1	O26C—C25C—H25H	109.5
C9B—C8B—C13B	116.1 (3)	H25G—C25C—H25H	109.5
C9B—C8B—C7B	122.8 (3)	O26C—C25C—H25I	109.5
C13B—C8B—C7B	121.0 (3)	H25G—C25C—H25I	109.5
F16B—C9B—C10B	118.8 (3)	H25H—C25C—H25I	109.5
F16B—C9B—C8B	117.7 (3)	C23C—O26C—C25C	118.7 (3)
C10B—C9B—C8B	123.5 (3)	O1S1—C1S1—O2S1	126.7 (4)
C9B—C10B—C11B	119.1 (4)	O1S1—C1S1—H1S1	116.6
C9B—C10B—H10B	120.4	O2S1—C1S1—H1S1	116.6
C11B—C10B—H10B	120.4	O1S2—C1S2—O2S2	127.1 (4)
C10B—C11B—C12B	119.5 (3)	O1S2—C1S2—H1S2	116.4
C10B—C11B—H11B	120.2	O2S2—C1S2—H1S2	116.4
C12B—C11B—H11B	120.2	O2S3—C1S3—O1S3	127.1 (4)
C13B—C12B—O15B	115.5 (3)	O2S3—C1S3—H1S3	116.5
C13B—C12B—C11B	119.9 (3)	O1S3—C1S3—H1S3	116.5
O15B—C12B—C11B	124.5 (3)		

### Hydrogen-bond geometry (Å, °)

**Table:**

*D*—H···*A*	*D*—H	H···*A*	*D*···*A*	*D*—H···*A*
N1A—H1A1···O2S3	0.92	1.84	2.750 (4)	172
N1A—H1A2···O2S1^i^	0.92	1.83	2.737 (4)	167
N1A—H1A2···O1S1^i^	0.92	2.60	3.314 (4)	135
N1B—H1B1···O1S3	0.92	1.83	2.729 (4)	166
N1B—H1B1···O2S3	0.92	2.61	3.329 (4)	136
N1B—H1B2···O1S1	0.92	1.84	2.756 (4)	173
N1C—H1C1···O2S2^ii^	0.92	1.85	2.743 (4)	165
N1C—H1C1···O1S2^ii^	0.92	2.61	3.330 (4)	135
N1C—H1C2···O1S2	0.92	1.82	2.733 (4)	173

Symmetry codes: (i) *x*+1, *y*, *z*; (ii) *x*+1/2, −*y*+3/2, −*z*+1.
